# Mechanical Recyclability of TPS/PVA Blends and Their Comparison with Other Bioplastics

**DOI:** 10.1021/acsomega.5c06947

**Published:** 2026-03-06

**Authors:** Noelia Martínez-Pérez, Juan C. García Quesada, Ignacio Martín-Gullón, Iluminada Rodríguez-Pastor

**Affiliations:** Institute of Chemical Process Engineering, University of Alicante, P.O. Box 99, 03080 Alicante, Spain

## Abstract

Thermoplastic starch-PVA blends currently present great potential since they show stable mechanical performance along with the advantage of compostability and self-biodegradability in the marine environment. However, one step further could be attained if this ocean-friendly compound could be mechanically recycled and then promote circular economy applications before its final composting. The main goal of this work is to study, for the first time, the mechanical recyclability of TPS/PVA compounds up to ten cycles, using blends based on potato, wheat, and cassava starch. Furthermore, the results are compared to those obtained by reprocessing commercial PLA and PHB compounds. As the mechanical reprocessing cycles proceed, all TPS/PVA compounds retain the mechanical performance after ten cycles, and especially toughness is also maintained. At the same time, there is evidence of some chain scission in starch, observed in earlier cycles for cassava-based compounds, although it is ultimately superior in a potato mixture. Interestingly, the starch syneresis progress as well as some chain scission allows a stable melt-flow index, up to cycle 10. In addition, wheat compounds could have the highest recycling potential, with less chain scission and syneresis, being able to redistribute the plasticizer in the starch-rich phase, improving its plasticization. Regarding commercial biopolymers, PLA loses toughness dramatically from cycle 4 and PHB from cycle 1, and the melt-flow index increases sharply, denoting a significant polymer degradation and poor recyclability potential.

## Graphical abstract

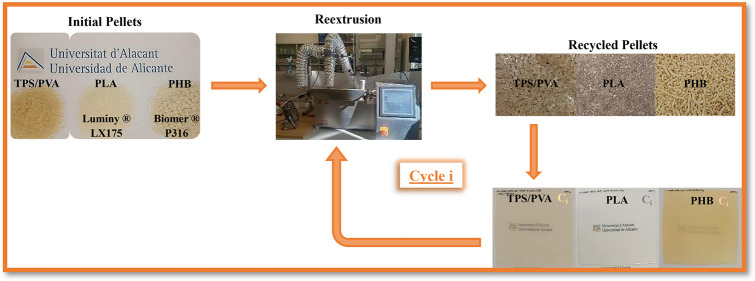

## 1 Introduction

The development of petroleum-sourced plastic materials has brought about a revolution in the 20th century society, finding application in almost all fields of daily life. Later, biobased plastics were developed several decades ago when concerns were raised about price oscillation and even shortage of raw materials. Nonbiodegradable biobased plastics equivalent to petroleum-based ones (like biopolyethylene^1^) appeared first on the market, followed by (i) new biobased plastics with compostable properties, such as polylactic acid (PLA),^2,3^ (ii) those based on biopolymers like polyhydroxyalkanoates (as poly-3-hydroxybutyrate, PHB),^4,5^ and lately (iii) compostable and petroleum-based plastics with similar properties to nonbiodegradable ones like polybutylene adipate terephthalate (PBAT).^6^ Concern has focused more on the pollution caused by uncontrolled disposal of plastic materials and their effect on oceans and aquatic life due to microplastics.^7,8^ However, plastic compostable in industrial conditions does not fully solve aquatic pollution in the form of microplastics. Thermoplastic starch (TPS) is self-biodegradable and compostable at both industrial and home conditions and could be a solution for aquatic pollution, but current commercial TPS is blended with nonbiodegradable polymers.^9^

According to challenges 12–15 of the UN 2030 Sustainable Development Agenda, all materials must be designed to have other uses after their end-of-life to promote a circular economy, avoiding single-use items even though these are fully biodegradable. Nonbiodegradable thermoplastics are thermally stable and present good expectations for both chemical and mechanical recycling, and there is high progress in the market with plastic recycled items (even though they are not a solution to environmental pollution).^10,11^ However, biodegradable bioplastics were designed and compounded to be specifically composted and/or self-degraded after use, without any objective of recyclability.^12^ Nowadays, there is a growing and urgent interest in studying the potential chemical or mechanical recycling of biobased and biodegradable polymers, mostly focused on PLA, which shows severe difficulties due to thermal degradation when reprocessing,^13^ as shown below. There are scarce studies on both PHB and TPS recyclability, although the production of the latter is currently on the rise, blended with other compostable or biodegradable polymers, since as a stand-alone, this compound lacks stability in properties.^9^

Regarding PLA mechanical recycling, there are several papers, but only a few are based on twin-screw extrusion without the addition of reinforcements or additives. There is a general agreement that after only 3–5 reprocessing cycles, there is an important decrease in mechanical performance in either tensile strength or elongation at break, increasing brittleness, due to molecular weight decrease by degradation and increase of internal porosity.^14–17^ Only Zenkiewicz et al.^18^ reported mechanical consistency up to 10 cycles, arguing that molecular weight decrease yielded an increase of crystallinity compensating stability. García et al.^19^ analyzed the effect of reprocessing wet or dried PLA, pointing out that dried samples only suffered thermo-mechanical degradation, whereas nondried samples also suffered hydrolysis, induced by the presence of moisture, which affects the ester groups.

Concerning the mechanical recycling of PHB through extrusion, there are very few references. There is an agreement that PHB can be reprocessed only 2–3 times, with a drop in mechanical properties and thermal stability and an increase in the crystallinity of the samples in cycle 2 due to degradation by chain scission, which generates chemocrystallization.^20,21^ By contrast, Plavec et al.^22^ reached 11 recycling cycles working with PLA/PHB blends, keeping constant tensile properties and thermal stability, but with an important decrease in the viscosity and elongation at break of the samples, indicating a lower molecular weight caused by thermal degradation, which was also observed by Farias et al.^23^ after 5 recycling cycles.

In terms of mechanical recycling through the extrusion of TPS, few articles were found. All of them were related to compound blends with other polymers, mostly of nonbiodegradable and fossil-based origin. Tavanaie et al.^24^ reported 5 cycles of mechanical reprocessing of TPS/PP blends without significant TPS degradation, showing in further cycles some decrease in viscosity and tensile properties. Similar conclusions were obtained by Peres et al.^25^ analyzing HDPE/TPS blends, with stable properties for 10 cycles. Mantia et al.^26^ studied the reprocessing of polycaprolactone (PCL)/TPS blends for 5 cycles, with a decrease in the impact test in cycle 1 and an increase in the tensile modulus after cycle 3, possibly due to cross-linking in the starch phase, as well as an increase in PCL phase crystallinity by reduction of its molecular weight. Oliveira et al.^27^ studied the mechanical recycling of PP/PBAT/TPS blends during 7 cycles, observing clear degradation, higher stiffness, and a drop in impact strength due to the reduction of PBAT/TPS domains by degradation, resulting in better PP compatibility. In this context, it should be mentioned that caramelization occurs when starch is heated up during recycling, yielding degradation and dark-colored films due to condensation reactions between the small molecules obtained from the degradation process and the macromolecular chains, as reported by different authors.^28–30^ In addition, in between each reprocessing cycle, starch syneresis and retrogradation can be accelerated,^31^ resulting in a loss of mechanical performance by producing brittleness.

It is important to mention that no previous literature report regarding the mechanical recycling of TPS/PVA was found. Although PVA has a petrochemical origin, it is biodegradable under a widespread variety of bacteria in both aerobic and anaerobic conditions, and self-biodegradability is effective in marine water environments as well as in papermill wastewater treatment.^32^ Additionally, TPS promotes and accelerates the PVA biodegradation in aquatic environments when forming TPS/PVA blends.^33,34^ As a result, TPS/PVA blends not only exhibit a highly stable compounded system,^35,36^ thanks to the excellent compatibility among the polymers—which leads to improved mechanical properties—but also pose no threat to marine ecosystems. Consequently, interest in commercializing these ocean-friendly bioplastics is steadily increasing.

The objective of the present paper is to study the mechanical recycling of these ocean-friendly TPS/PVA blends, pointing out the focus of the effect of starch from different botanical origins. Three different compounds, TPS/PVA, were produced by a twin-screw extruder from wheat (A-type crystallinity), potato (B-type), and cassava (C-type) starch. Initial TPS/PVA compounds were thermoformed and further subjected to consecutive cycles of twin-screw extrusion and thermoforming, with a full characterization to analyze their mechanical recycling potential. For comparison purposes, results concerning the recycling processing (under the same conditions) of commercial PLA and PHB products are also shown.

## 2 Materials and Methods

### 2.1 Materials

Potato, wheat, and cassava starch were supplied by Emsland (Emlichheim, Germany), Sigma-Aldrich (Madrid, Spain), and a local supplier (Alicante, Spain), respectively. PVA 20–88 (viscosity (mPa·s) – hydrolysis degree (mol/%), Mw: 20,000–30,000) was purchased from Sigma-Aldrich (Madrid, Spain), glycerol was purchased from Fisher Chemical (Geel, Belgium), and zinc stearate was purchased from Sigma-Aldrich (Madrid, Spain). All chemicals were used without further purification. Commercial PLA is the reference Luminy LX175 from TotalEnergies Corbion (Gorinchem, The Netherlands). Commercial PHB is the reference Biomer P316 from Biomer (Schwalbach, Germany).

### 2.2 TPS/PVA Blend Preparation

Different TPS/PVA compounds were prepared by using, alternatively, potato, wheat, and cassava starch. Initially, a 50:50 mixture of starch and PVA was placed in a mixer, then water (40 phr) and glycerol (60 phr) were added (phr related to total polymers) and mechanically mixed. The blend was placed in a ZIP bag and kept in an oven for 17 h at 70 °C to favor the polymer swelling process and obtain a homogeneous mixture. Subsequently, zinc stearate (0.5 wt %) was added and mixed. Finally, the mixture was fed to a twin-screw corotating extruder (Thermo Scientific Process 11) at 200 rpm, with a temperature profile from 100 to 200 °C (feed to die) and subsequently pelletized, forming cycle 0 pellets.

### 2.3 Biopolymer Mechanical Recycling

To simulate the transformation processing, 1 mm thick films of each TPS/PVA compound were formed by compression molding in a hot-plate press at a pressure of 7 tons for 10 min at 160 °C. Films of PLA and PHB were also produced from the corresponding raw pellets, following a similar process, setting the temperature to 170 and 180 °C, respectively. Finally, the films were cooled for 5 min. The obtained TPS/PVA and PHB films were cut, and the PLA films were ground. The resulting materials were reprocessed in the twin-screw extruder, producing reprocessed pellets, thus completing a recycling cycle (Scheme 1). This complete recycling process was repeated successively (cycle i = C_i_), for 10 cycles for TPS/PVA compounds, considered enough at a practical level. For PLA, only 7 cycles were possible to carry out due to a sharp decrease in viscosity, which made reduce the die temperature to 190 °C in cycles 4–5 and 175 °C in cycles 6–7. Only 5 cycles were possible for PHB due to the impossibility of further reprocessing because of its degradation.

**Scheme 1:**
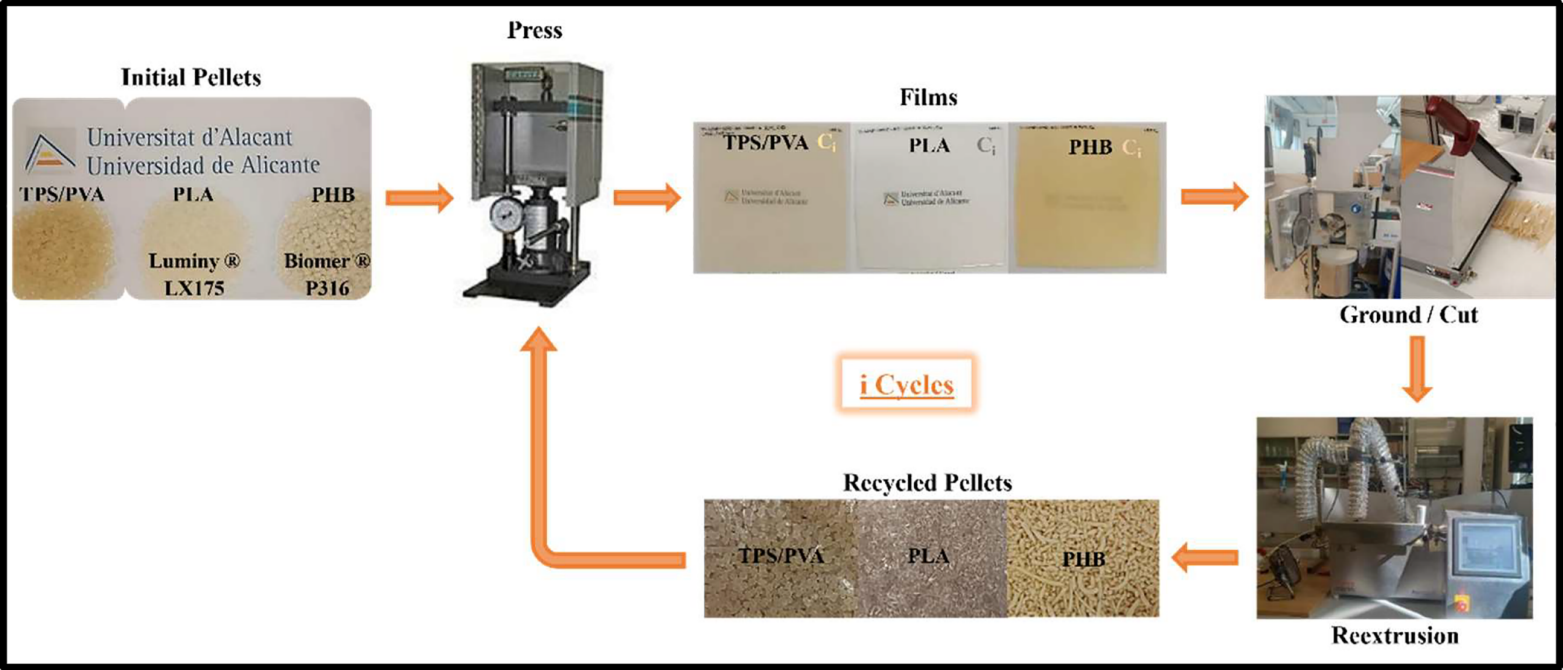
Mechanical Recycling Process

### 2.4 Characterization

Potato, wheat, and cassava raw starch materials were characterized by amylose-amylopectin ratio, grain morphology, particle size, porosity, and crystalline structure. Amylose and amylopectin content were quantified in duplicate using the concanavalin-A precipitation method with an amylose/amylopectin assay kit, as described elsewhere.^37^ Morphology of the starch grains was explored through scanning electron microscopy (SEM) images, using a Hitachi S3000N, with an accelerating potential of 15 kV. The particle size distribution of starch grains was carried out by the laser diffraction (LD) technique (Malvern Instruments, model 2000, Worcestershire, UK) in duplicate, as described elsewhere.^38^ Starch internal pore measurements were performed using a mercury porosimeter POREMASTER-60 GT (Quantachrome Instruments, USA) to determine the pore size distribution (PSD), in duplicate. A forced mercury pressure range of 0.0036–413 MPa was applied to carry out the measurements, corresponding to the pore radius between 0.0015 and 173 μm, allowing the detection of both mesopores and certain starch macropores, assuming a cylindrical shape, according to the Washburn equation.^39^ Before the pore measurements, samples were dried in an oven at 110 °C for 5 h and degassed in vacuum.

Color analysis of bioplastic reprocessed films was characterized by applying the CIELab color space method to pictures taken in a controlled light chamber. Basically, *L* indicates lightness and its range is from black to white (0 to 100), and *a* and *b* are chromaticity coordinates, indicating *a* the green to red range (−120 to 120) and *b* the blue to yellow range (−120 to 120). A script file in the MATLAB R2022a environment was written and used to determine the parameters of the three coordinates cited along an area of each sample film picture (around tens of thousands of pixels), and average values and standard deviations of the pixels were analyzed.

Melt-flow index (MFI) of all the studied bioplastic samples was performed using a METROTEC equipment model MFI – 500/AP by TECHLABSYSTEMS (Lezo, Spain), following the ASTM D1238 standard and the gravimetric method (at least in triplicate). TPS/PVA samples were characterized at 175 °C and 5 kg load, PLA samples at 190 °C, and PHB samples at 180 °C, both with 2.16 kg load. All samples were dried at 80 °C for 30 min to ensure the same humidity conditions.

For the following characterization techniques, potato, wheat, and cassava-based TPS/PVA films were conditioned in a glovebox with controlled relative humidity of 50% for 48 h and 23 °C prior to their further characterization:

- The crystalline structure of either raw starches, PVA and TPS/PVA films, was determined by X-ray diffraction on a Bruker diffractometer (D8-Advance model, Ettlingen, Germany) equipped with a KRISTALLOFLEX K 760-80F X-ray generator (power = 3,000 W, voltage = 20–60 kV, and intensity = 5–80 mA), which has an X-ray tube with a copper anode (λ = 1.54056 Å). The equipment operated at 40 kV and 40 mA with 2θ varying from 2.5° to 50° with a step size of 0.05° for the raw starch materials, and from 5° to 35° with a step size of 0.5° for the pure PVA and TPS/PVA films.
- Moisture content of 1 × 1 cm^2^, expressed as a percentage (grams of water in 100 g of sample), was calculated by quintuplicate, through the difference between the sample initial mass (*m*_0_) and its mass after drying at 110 °C for 5 h (*m*_1_).
- The viscoelastic properties were studied by dynamic thermo-mechanical analysis (DTMA) in a METTLER-TOLEDO DMA 1 in single cantilever mode, from −100 to 60 °C with a 3 °C/min heating ramp. These resulting curves were smoothed.
- Tensile properties (Instron 3344 equipped with a 2000 N load cell, following the ASTM D882) were determined for all the studied TPS/PVA and PLA resulting films (at least by quintuplicate); flexural test (same equipment with 2810-400 bend fixture, following ASTM D790) was done to PHB ones (at least by quintuplicate).
- The oxygen transmission rate (OTR) test was also carried out for TPS-based samples, PLA and PHB ones, at least by duplicate, using a Systech oxygen permeation analyzer model 8500 from Metrotec, following the ASTM D3985. Pure oxygen (99.9%) was introduced into the upper half of the diffusion chamber while nitrogen was injected into the lower half, where an oxygen sensor was located. Film samples were previously conditioned in the described glovebox (RH = 50%) at room temperature for 24–48 h prior to testing. Film thickness was determined by a digital micrometer at 8 different positions to ensure trustable values.

## 3 Results and Discussion

### 3.1 Starch Grain Characterization

Concerning **amylose and amylopectin** content, results obtained revealed that wheat has the highest amylose content (26 ± 2%, 74 ± 2% amylopectin), followed by cassava (19 ± 2%, 81 ± 2% amylopectin) and potato (16 ± 1%, 84 ± 1% amylopectin). These results are in good agreement with previous works in the literature,^40,41^ where legumes regularly present the highest amylose content, cereals middle values, and tubers the lowest values of amylose.

Regarding the morphology of the initial grains, Table 1 shows the starch **particle size** determined by LD (see Supporting Information, Table S.1) in terms of D50 and D10, showing the smallest particle size for cassava, whose D10 is below 1 μm. These values are in agreement with those observed with **SEM** (Figure 1). SEM pictures also show that potato starch granules are oval-shaped and clearly the largest, wheat has a lenticular shape, and cassava starch grains are rounded/polyhedral, with the smallest and narrowest size distribution. Shape is important because it yields different void (or interparticle) spaces according to different packings, affecting plasticizer diffusion in between the starch grains,^42^ developing the swelling and gelatinization processes, and in turn, its processability and plasticization.

**Figure 1:**
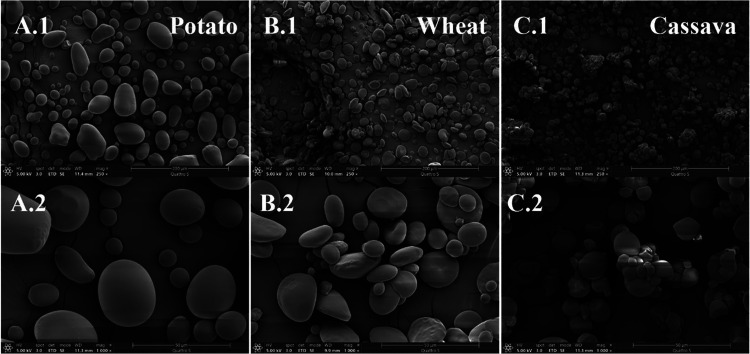
SEM micrographs of potato (A), wheat (B), and cassava (C) starch at 250x (1) and 1000x (2).

**Table 1:** Starch Grain Properties According to Parameters Obtained by the LD Technique: D50 and D10, and to Parameters from Mercury Porosimetry: Total Hg-Intruded Volume, Hg-Intruded Volume for Pores Below 500 nm (Intraparticle Pore Volume), and Average Interparticle Void Space^a^

botanical origin	average particle size (D50) (μm)	D10 particle size(μm)	total Hg-intruded volume(mm^3^/g)	average interparticle pore space (μm)^b^	Hg-intraparticle pore volume (mm^3^/g)
potato	44.79 ± 0.01	24.7 ± 0.1	481.5 ± 0.6	13.5 ± 0.9	4.2 ± 0.6
wheat	20.04 ± 0.07	12.3 ± 0.1	78.0 ± 0.5	3.9 ± 0.6	19.6 ± 0.5
cassava	13.65 ± 0.06	8.6 ± 0.7	786.4 ± 0.6	7.9 ± 0.7	3.8 ± 0.6

a The errors represent the standard deviation of the average of two independent replicates.

b Results in terms of distributions statistical mode.

Mercury porosimetry was carried out to determine both the void space between the grains (interparticle space) and the internal **porosity** (intraparticle space), and the results are shown in Figure S.1, where the plot shows the derivative intruded volume vs pore size. Table 1 shows that Hg-interparticle void sizes are slightly below the D10 values determined by LD, which validates the results of both techniques. However, it is important to point out that total Hg-volume intruded is considerably lower for wheat (10 and 20% of those of cassava and potato, respectively), indicative of the importance of the starch granules' shape and their packing degree, which may greatly affect the initial effective diffusion of plasticizers into the grains.

Wheat presents markedly the highest intraparticle pore volume (around 20 mm^3^/g vs 4 mm^3^/g of tuber-based ones), especially in the range of the mesopores (between 2–50 nm, see Figure S.1). These results are in agreement with those shown by Sujka et al.,^43^ who determined the internal porosity by means of the specific surface area through 77 K nitrogen adsorption isotherms, with the same tendency: both potato (0.11–0.243 m^2^ g^–1^) and tapioca (0.280 m^2^ g^–1^) exhibited very similar and remarkedly lower values than wheat (0.534 m^2^ g^–1^).

On the other hand, and in contrast, wheat also presents, more notably, the lowest interparticle pore volume, whose diameter is concentrated between 1 and 5 μm (Figure S.1), compared to 1–20 μm for cassava and 5–20 μm for potato. Therefore, in addition to having a lower total volume of interparticle porosity, these wheat starch pores are much smaller than those of tubers.

Thus, in view of the results obtained, it is possible to state that wheat might show a lower external diffusion (lowest interparticle pore volume), resulting in lower kinetics for the starch initial disaggregation and swelling. However, it also reveals higher internal diffusion (highest intraparticle pore volume), which promotes more effective gelatinization.

### 3.2 Visual and Color Characterization

Regarding **visual properties** (Figure 2), it must be stated that all the TPS/PVA reprocessed films maintain their homogeneity constant with the reprocessing cycles, increasing their color. Furthermore, transparency seems to increase for both potato and wheat films but decreases for cassava TPS/PVA samples. The color of commercial PLA and PHB obtained films also increases, while their transparency and homogeneity decrease and, in terms of handling, their brittleness increases.

**Figure 2:**
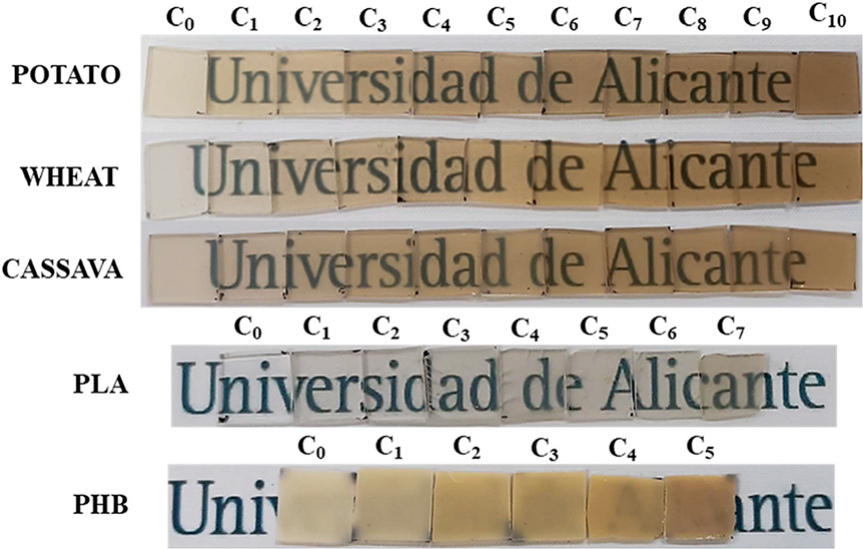
Obtained samples for the mechanical recycling process of potato, wheat, cassava starch, commercial PLA, and commercial PHB.

**CIELab** analysis carried out on all the groups of samples for the different bioplastics present the same tendency with the recycled round, as can be seen in Figure 3, with a decrease of *L* (whiteness) and an increase of *a* (redness) and *b* (yellowishness). The decrease in *L* was abrupt for PHB and, to a relatively minor extent, for PLA, whereas there was a sharper increase of *a* for PHB. These results are in agreement with previous works: Yang et al.^44^ reported an increasing dark brown color during PHB processing associated to the formation of oligomers with crotonate end groups together with a drop in molecular weight, whereas Mysiukiewicz et al.^45^ observed, when reprocessing PLA, a decrease in viscosity and yellowing, and a reduction of the polymer molecular weight by the thermooxidative degradation suffered during twin-screw extrusion. TPS/PVA compounds also show a decrease in whiteness, but to a minor extent with respect to commercial bioplastics, except for the case of potato blends. With respect to color evolution, there is no special difference for different origin starch-based compounds versus the reprocessing cycle, slightly increasing redness and yellowishness. These color changes could reflect the development of caramelization reactions^28–30^ similar to Maillard reactions, which involve amino acids and carbohydrates. In any case, when comparing the behavior of different starch samples, a prominent darkening and, hence, degradation of potato specimens is readily noticeable with respect to cassava and wheat starches.

**Figure 3:**
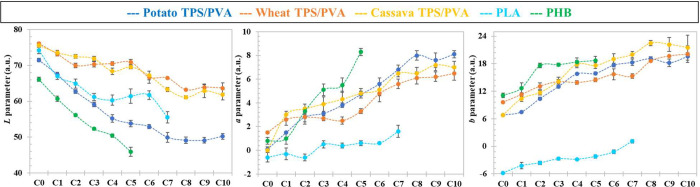
CIELab parameters resulting from TPS/PVA, PLA, and PHB films are shown in Figure 2. The error bars are provided by the MATLAB script and represent the standard deviation of the average color of the analyzed pixels.

### 3.3 Rheology Characterization

Since polymer molecular weight is directly proportional to their viscosity, MFI can be a good tool for qualitative estimation and comparison of the polymers' molecular weight. Figure 4B shows an abrupt increase in the MFI for both PLA and PHB, indicative of a high decrease of viscosity when reprocessing, which matches the degradation observed by the color analysis (due to molecular weight reduction by chain scission). By contrast, starch samples (Figure 4A) present MFI values of the same order of magnitude along the reprocessing cycles, reflecting structural integrity that is not highly compromised. However, the observed trend is relatively complex and is very probably the result of the superposition of different contributions.

**Figure 4:**
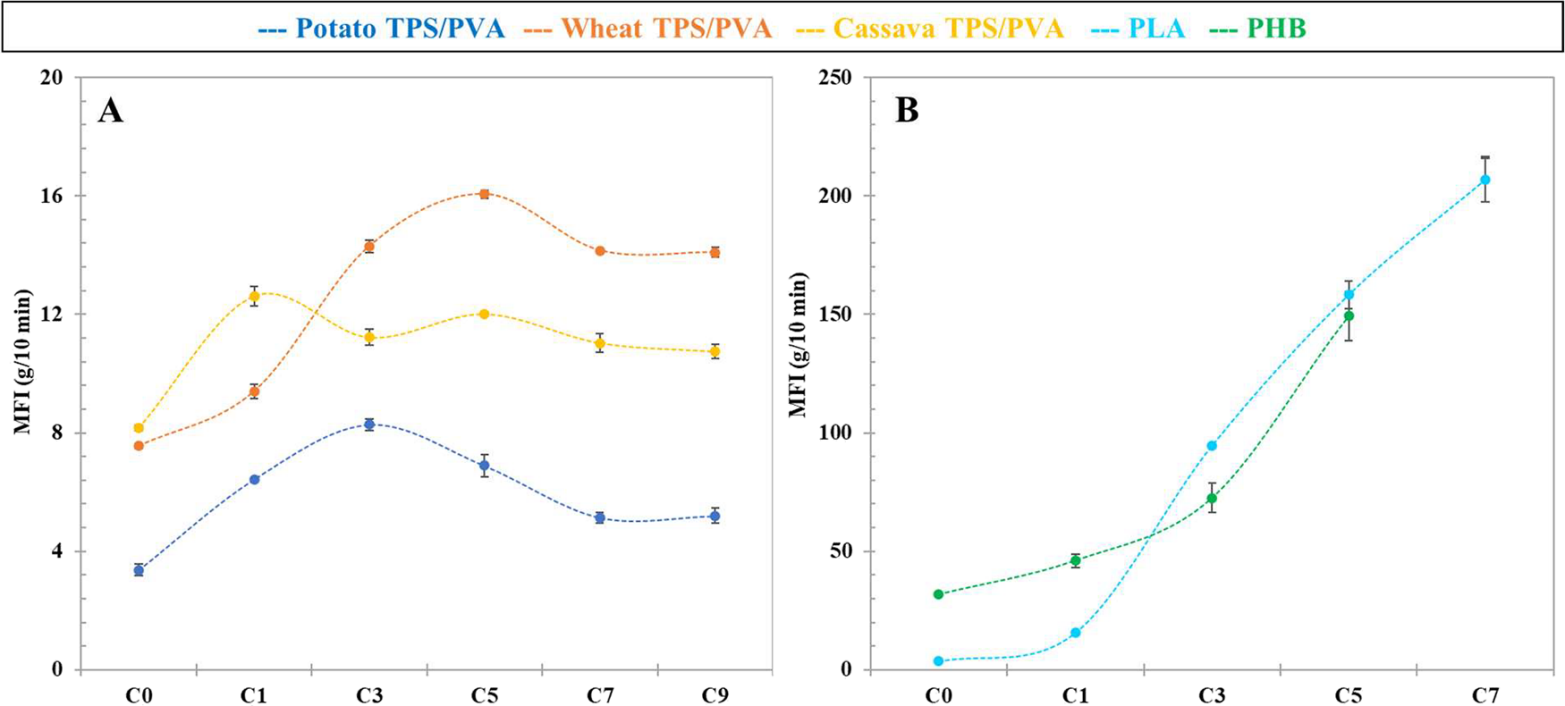
Melt-flow index of potato, wheat, and cassava TPS/PVA blends (A) and commercial PLA and PHB (B). The error bars represent the standard deviation of the average of at least three independent replicates.

In terms of the three initial TPS/PVA films (C_0_), the potato-based blend has the lowest value (3.4 g/10 min), followed by wheat (7.56 g/10 min), and very close to this is cassava (8.17 g/10 min). These results can be related to the starches' amylose-amylopectin content and their porosity. Since potato starch presents the highest amylopectin content, it results in a polymeric matrix structurally more compact due to the branching and the higher presence of intermolecular H-bonds, promoted by a higher number of oxygenated groups. Although cassava starch also presents a higher amylopectin content than wheat, it is markedly the most porous starch (highest total Hg-intruded volume, Section 3.1, Table 1) and may give rise to better processability and gelatinization, promoting the polymeric chain mobility. Moreover, wheat starch possibly has not been fully plasticized, as a consequence of its poor porosity, resulting in a higher viscosity than expected.

Regarding the MFI evolution, all samples present first a smooth increase, which is expected according to thermo-mechanical degradation (chain scission), with different top values for each starch-based compound: cycles C_0_ - C_1_ for cassava, C_0_ - C_3_ for potato, and C_0_ - C_5_ for wheat samples. Subsequently, there is a smooth decrease in MFI for a final stable value after 10 cycles, keeping the order of magnitude of C_0_. It might be highlighted that this degradation process seems to take place concurrently with a progressive plasticizer loss (syneresis), which would bring a decrease in MFI, because of a more compact matrix structure (increase of the average molecular weight), increasing its viscosity and decreasing its mobility. In this way, compensating for the effect provoked by chain scission results in the observed tendency.

At the same time, when comparing tuber starches with similar and relatively big intraparticle pore sizes, the lower MFI index of potato would provoke a more relevant shear degradation, readily observable by color development (Section 3.2, Figure 3), and an increase in MFI that is prolonged to cycle 3 in comparison to cycle 1 in cassava.

The better stability of wheat samples with respect to potato, in terms of color development and a higher MFI, reflecting a lower shear degradation, might indicate that other factors would be responsible for the progressive increase in MFI up to cycle 5. In this case, the hypothesis that will be supported by other techniques later (XRD, Section 3.4 and DMA, Section 3.6) consists of the fact that the smaller pore size in wheat granules (Section 3.1, Table 1) hinders the adequate swelling and initial plasticization of wheat starch, which require several reprocessing cycles (greater disruption of its granules) in comparison to cassava and potato starches. As a result of this prolonged plasticization of wheat starch, the mobility of the polymer chains is promoted, thereby reducing its melt viscosity.

### 3.4 Crystallinity Characterization

Figure 5 shows the **XRD** diffraction patterns of the different raw starches. Amylopectin governs the crystalline structure in the grains since amylose forms an amorphous phase. Amylopectin forms a double helix structure, forming crystallites of 10–20 glucose molecules stabilized by hydrogen bonds, which may conform to different polymorphs, A and B types, or their mixture known as C-type.^38,46^ Cereal starches, such as wheat, are usually characterized by pattern A (monocyclic structure), tuber starches such as potato by pattern B (hexagonal network), and certain roots like cassava show mostly C-type.^38,47–49^ A-type crystallinity presents XRD characteristic peaks at 2θ 12.5°, 15.3°, 17.0° (main), 18.1°, 20.0°, 23.2°, and 26.2°, whereas B-type pattern in 2θ 5.5°, 11.1°, 15.1°, 17.1° (main), 19.7°, 22.3°, 24.1°, and 26.3°. Figure 5 shows clearly that potato matches B-type and wheat A-type; meanwhile, the cassava curve shows certainly a mix of both types, although it is mostly of A-type. The raw PVA pattern, also shown in Figure 5, exhibits crystalline phase structures stabilized by hydrogen bonds,^50,51^ yielding peaks at 2θ 11.5°, 19.4° (main) and 22.7°,^52–54^ as found in this work.

**Figure 5:**
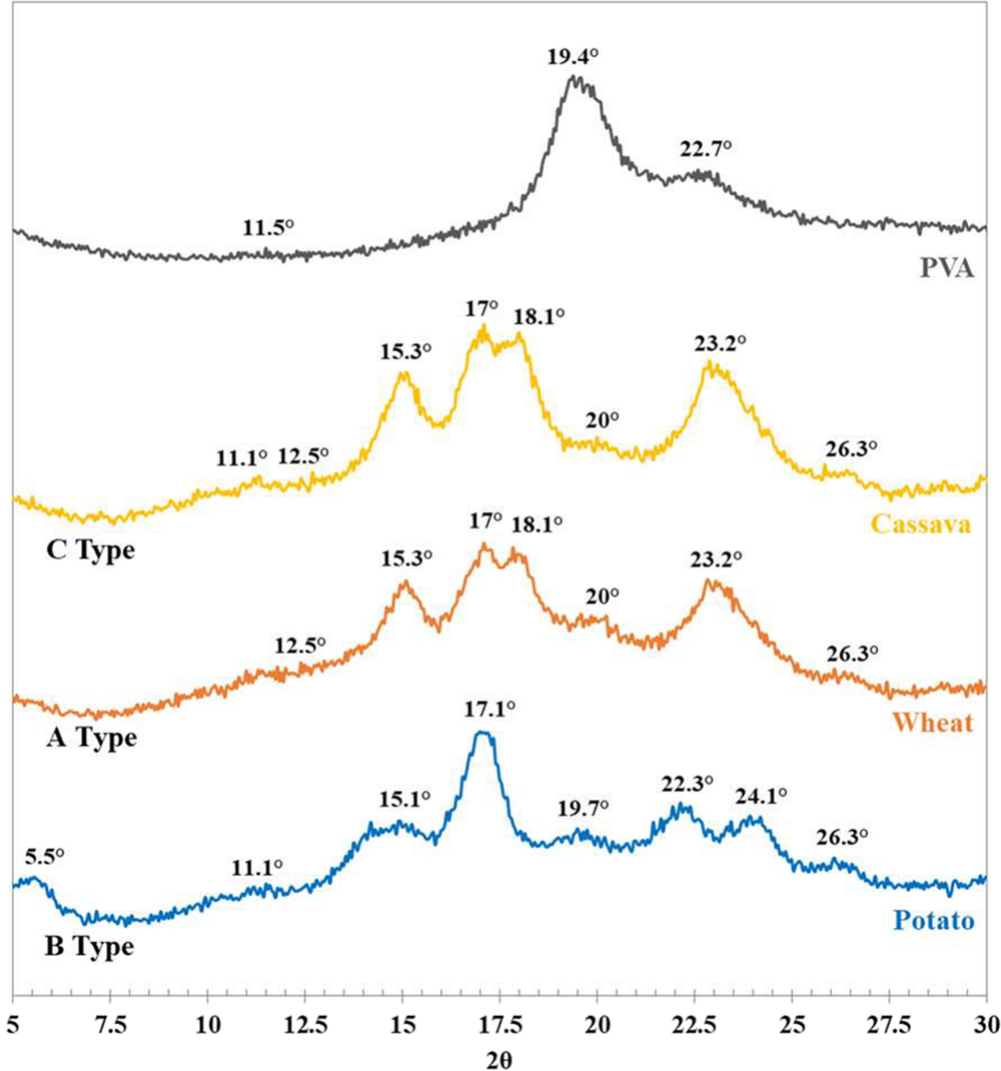
XRD of potato, wheat, and cassava starch grains and pure PVA.

Figure 6 shows the resulting XRD curves for each TPS/PVA initial sample (C_0_) and their evolution during reprocessing. By comparison with the Figure 5 curves, plasticization leads to a complete destruction of the parent grain crystallinity, e.g., peaks at 2θ 15.1°, 17°, 22.3°, 23.2°, and 24.1°, regardless of the starch origin, as stated in the literature.^37,38,40,55,56^ However, at 2θ 12.5°, a peak of residual crystallinity is observed for the three C_0_ samples, more marked for wheat-based film, indicative of its lower initial plasticization. Furthermore, this peak is lost during reprocessing, contrary to what is observed for potato and cassava starches, which retain it, supporting the hypothesis of prolonged starch plasticization during reprocessing cycles for wheat.

**Figure 6:**
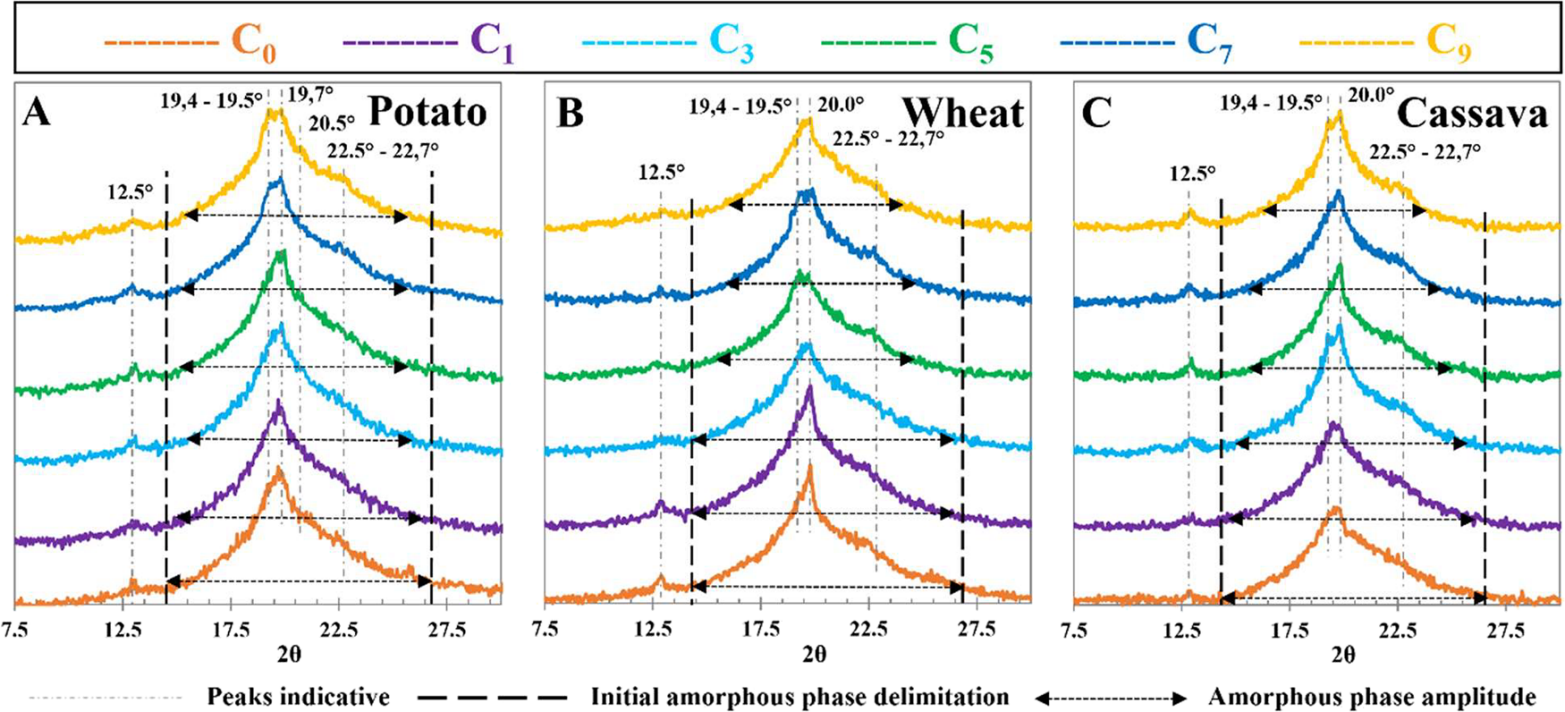
TPS/PVA films XRD diagrams of potato (A), wheat (B), and cassava (C) starch.

On the other hand, all the C_0_ TPS/PVA compounds present the main peak at 19.8–20.0°, which was not in the parent materials, and corresponds to the major peak of V-type crystallinity, which appears after the plasticization due to the retrogradation of the plasticized starch by the amylose-glycerol phase,^37,38^ which indicates that gelatinization took place. This peak is more pronounced for the wheat-based sample, as a consequence of its higher amylose content, which also has a small peak shoulder of the V-type system at 2θ 22.5°, indicative that retrogradation took place in wheat to a higher extent. V-type is also dominant for potato and cassava in C_0_, but not so well-defined as in the wheat sample.

Analyzing the evolution of potato film curves with the reprocessing cycles (Figure 6A), in terms of amorphous phase distribution, their crystallinity does not seem to vary after cycle 7, according to MFI results, where the native PVA peak (19.4°) emerges close to the V-peak (19.7°). This indicates a decrease in the V-type phase as a result of amylose chain scission, capable of partially hindering the crystalline retrogradation of the amorphous phase. Furthermore, the crystalline phase of PVA increases due to its recrystallization, caused by the loss of plasticizer. A similar effect is observed for wheat-based samples (Figure 6B), a decrease in V-type crystallinity until C_3,_ and a PVA peak emerging at C_5_. These results correspond with peaks observed for MFI. However, in this case, the decrease in V-type crystallinity could be related to the prolonged plasticization of wheat starch as a consequence of a more effective distribution of the plasticizer in the polymer network, forming more effective interchain interactions through H-bonds, preventing retrogradation. Cassava evolution along the cycles (Figure 6C) is less clear, although the PVA peak seems to emerge in C_1_ (matching MFI top) and subsequent changes toward a sharper V-type peak at C_5_, with crystallinity increasing for most of the process.

### 3.5 Hydration Characterization

The **moisture content** of the three TPS/PVA set of films is shown in Figure 7. It is reminded that prior to the moisture determination, all samples were conditioned in a glovebox at 23 °C and 50% relative humidity. C_0_ wheat-based film presents the highest value (20.2 ± 0.2% wt.), followed by cassava (19.4 ± 0.3% wt.) and potato (17.3 ± 0.1% wt.), similar to those reported by Luchese et al.,^47,55^ and slightly higher than those published in previous studies.^36,38,37^ There is a tendency with the amylose content of the starches (Section 3.1) and the V-type crystalline phase formation (Section 3.4, Figure 6). It is noted that most of the formulation water is lost during the extrusion-thermoformed process, and the moisture content is mainly due to water absorption, especially by glycerol-rich areas, which are mobile (syneresis) and hygroscopic. Therefore, results suggest that more crystalline domains within the films' amorphous phase (V-type crystallinity) promote segregation of glycerol-rich areas and then more absorbed water.

**Figure 7:**
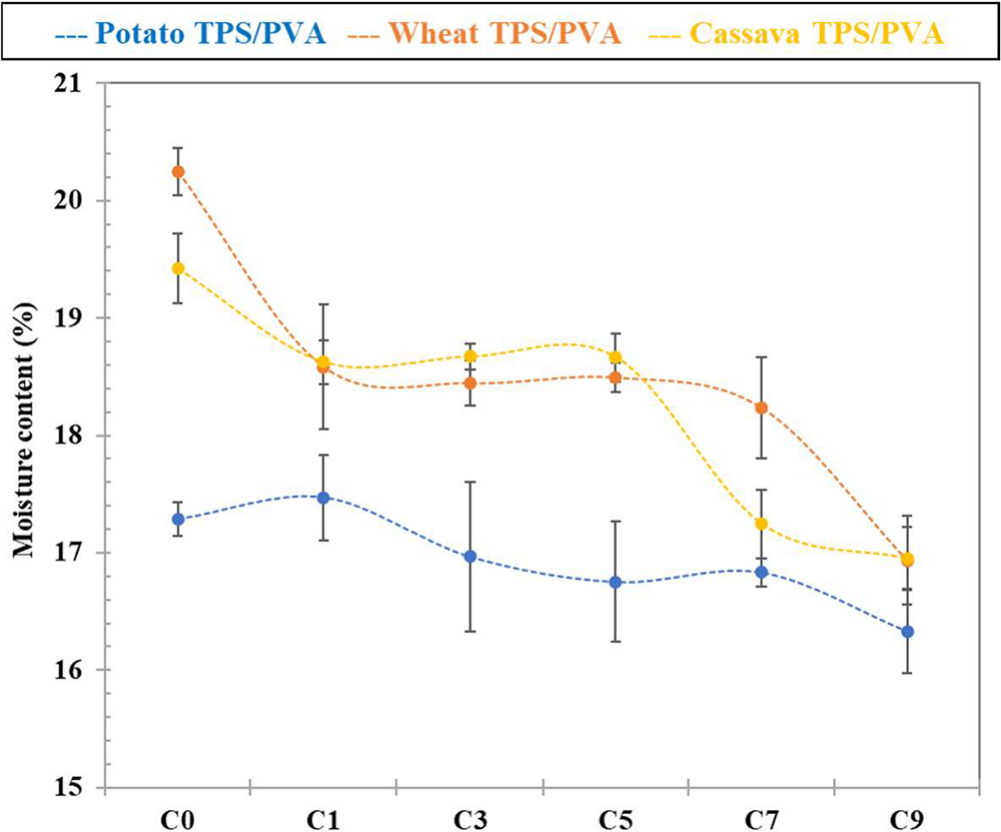
TPS/PVA films moisture content of potato, wheat, and cassava starch. The error bars represent the standard deviation of the average of five independent replicates.

On the other hand, the moisture content is decreasing with recycling cycles for the three sets of films, although not particularly significant (1–3 wt %), because of glycerol removal promoted by the thermo-mechanical applied processes during extrusion, resulting in a lower capacity to absorb moisture. It must be mentioned that this moisture decrease does not play a role in the evolution of the samples' viscosity by means of MFI (Section 3.3, Figure 4).

### 3.6 Mechanical-Dynamic Characterization

Concerning the **DMA** results, as can be observed in Figure 8, two relaxation processes are observed, similar to previous literature and attributed to a β–relaxation of a glycerol-rich phase, with a peak between −63 and −56 °C, and an α–relaxation associated with the segmental motion of starch chains, with a peak between −22 and −9 °C.^57,58^

**Figure 8:**
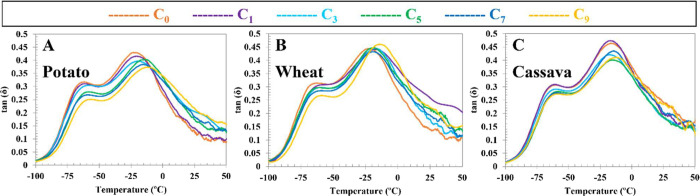
TPS/PVA film DMA spectra of potato (A), wheat (B), and cassava starch (C).

In general terms, it is possible to observe a shift of the maximum of these relaxations to higher temperatures and a decrease in the magnitude of the peaks with reprocessing, especially for potato- and cassava-based compounds, revealing a progressive decrease of plasticizer content in the glycerol-rich phase as well in starch-rich domains. It obviously indicates that glycerol is being lost with reprocessing cycles; meanwhile, starch undergoes a certain degree of degradation in view of color changes (Section 3.2, Figure 3). However, the wheat-based compound (Figure 8B) does not follow the previous trend for the α–relaxation peak, keeping peak temperature and intensity, except for C_9_, where both peak temperature and intensity increase, revealing a different process with respect to tuber-based compounds. These results can be due to the prolonged plasticization observed for wheat, in good agreement with MFI behavior (Section 3.3, Figure 4A) and the intraparticle pore size (Section 3.1, Table 1).

### 3.7 Mechanical Performance Characterization

Figure 9 shows the tensile property evolution along the reprocessing cycles for the three TPS/PVA blends. The first aspect to point out is the stability of the tensile properties along the reprocessing cycles, concluding that reprocessed TPS/PVA compounds are valid for the same applications.

**Figure 9:**
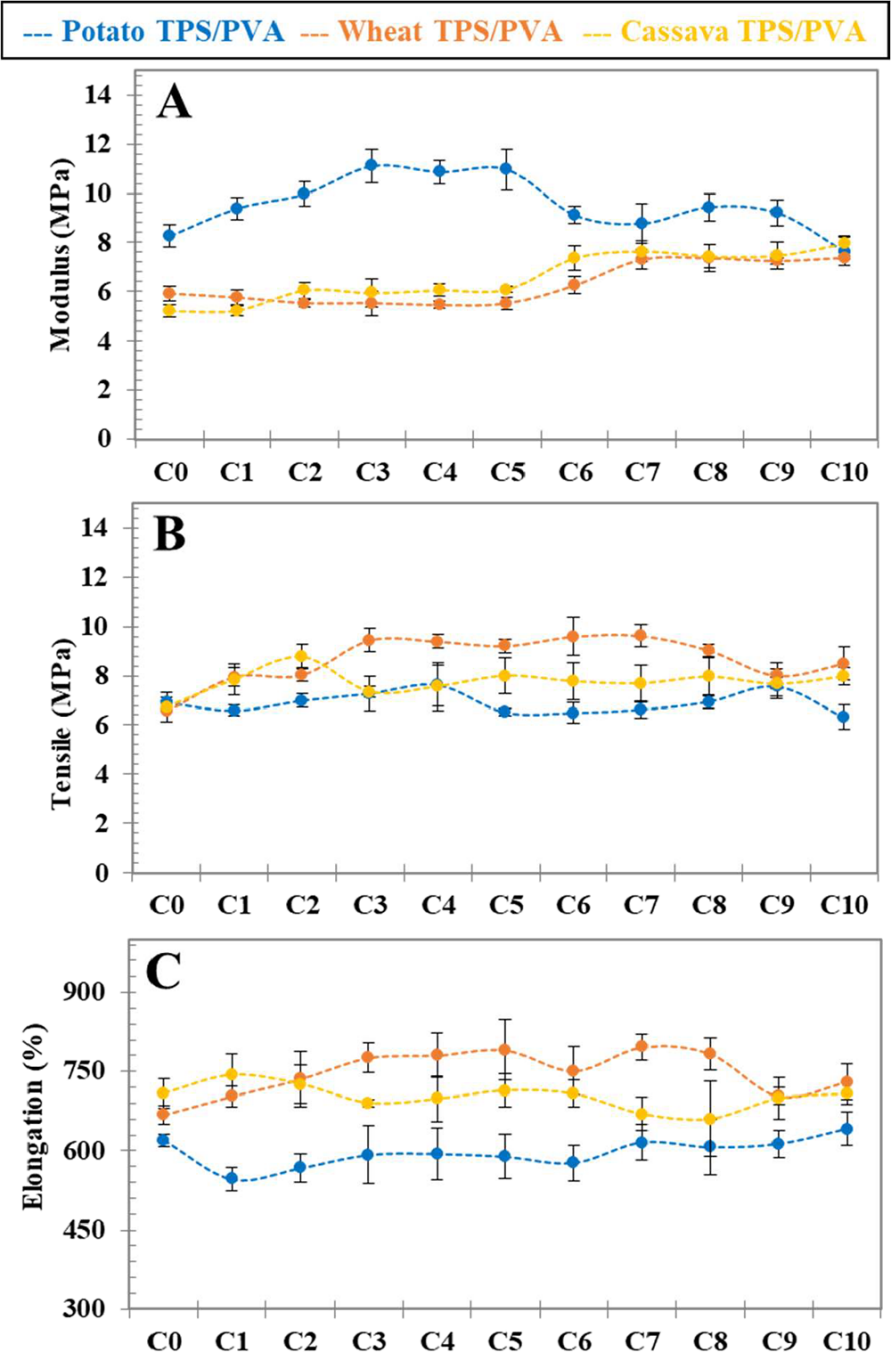
Mechanical properties of potato, wheat, and cassava TPS/PVA samples in terms of Young’s modulus (A), maximum tensile strength (B), and elongation at break (C). The error bars represent the standard deviation of the average of at least five independent replicates.

In relation to starch origin, potato starch-based compound increases slightly the Young’s modulus up to C_3_ (matching with MFI top value due to glycerol removal), a plateau up to C_5_ (a combination of glycerol removal and some starch chain scission), and a further slight decrease up to cycle 10 (advance degradation, as shown by DMA and color analysis). On the other hand, cassava- and wheat-based compounds show stable moduli up to C_5_ and a slight increase up to C_7_, with ongoing stability up to C_10_, indicating a more stable polymer network than that of the potato one. In addition, wheat starch-based compounds show increasing values of both tensile strength and elongation upon increasing reprocessing cycles, which is in agreement with the observed prolonged plasticization behavior structured by MFI (Section 3.3, Figure 4), XRD (Section 3.4, Figure 6), and DMA (Section 3.6, Figure 8) analysis.

Regarding the comparison with other commercial bioplastics, TPS-based ones are more stable with respect to PLA and PHB, whose properties are markedly deteriorated, as shown in Figure 10. PLA (Figure 10A), the stiffest of the studied bioplastics in the present work, loses toughness dramatically from cycle 4 and reaches complete brittleness after cycle 5, in agreement with previous works^14,15,17,18,59^ due to severe thermo-mechanical degradation^14,18^ and shown before in MFI analysis (Section 3.3, Figure 4B). In terms of PHB (Figure 10B), the most brittle of the bioplastics studied, Young’s modulus decreases sharply in C_1,_ whereas tensile strength smoothly decreases with reprocessing, similarly to results obtained by Rivas et al.^20^ and Main et al.,^21^ due to polymeric chain scissions and also in agreement with MFI results (Figure 4B).

**Figure 10:**
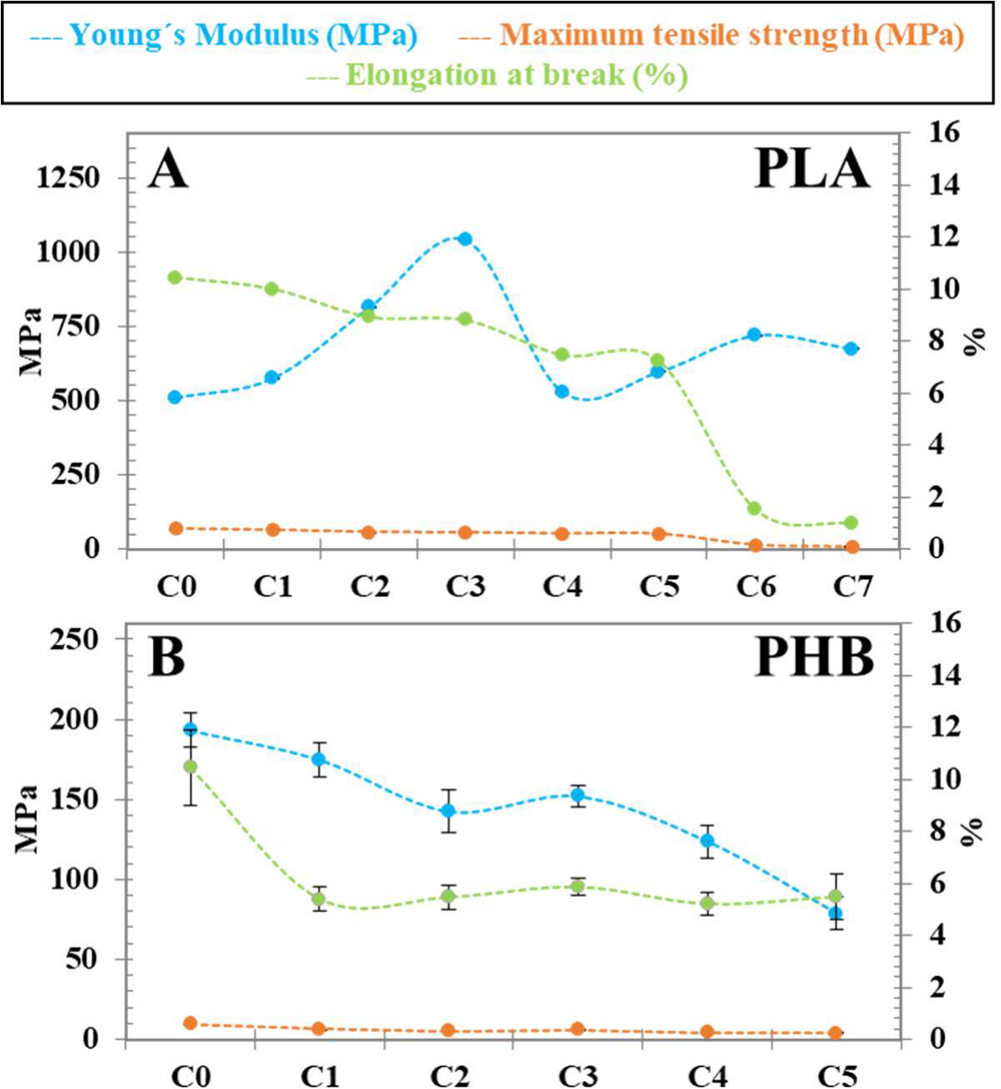
Mechanical properties of commercial PLA (A) and PHB (B) films. The error bars represent the standard deviation of the average of at least five independent replicates.

It is worth mentioning that only TPS/PVA blends do not lose toughness, keeping their properties above the initial values. In contrast, commercial PLA and PHB have very low recyclability limits in terms of mechanical performance. In addition, all the obtained TPS/PVA blends have tensile and elongation properties comparable to those of commercial low-density polyethylene (LDPE).^60^

### 3.8 Permeability Characterization

Barrier properties have been studied through OTR, and the results for TPS/PVA C_0_ and C_10_ samples are shown in Table 2 (for PLA and PHB, the other reprocessed cycle was studied due to the difficulty of producing valid films). It should be noted that TPS/PVA blends exhibit significantly lower OTR values than both PLA and PHB, thanks to the TPS/PVA being more polar and hydrophilic matrices. Since the commercial biopolymers have higher hydrophobicity and lower polarity, they enhance the oxygen affinity and, hence, their diffusion across the polymeric matrices, as reported by Khan et al.^61^ In this way, TPS materials have proven to be more competitive for applications where the products have relevant oxygen sensitivity.

**Table 2:** Potato, Wheat, and Cassava TPS/PVA Blends and Commercial PLA and PHB OTR^a^

starch botanical origin	recycling cycle	OTR (cm^3^·mm/m^2^·day)	average film thickness (mm)	commercial biopolymer	recycling cycle	OTR (cm^3^·mm/m^2^·day)	average film thickness (mm)
potato	C_0_	3.0 ± 0.2	1.04 ± 0.01	PLA	C_0_	23 ± 1	0.98 ± 0.01
	C_10_	4.6 ± 0.4	0.96 ± 0.01		C_4_	16 ± 5	0.92 ± 0.01
wheat	C_0_	2.8 ± 0.1	1.11 ± 0.01	PHB	C_0_	22 ± 2	0.91 ± 0.01
	C_10_	4.2 ± 0.3	1.09 ± 0.01		C_1_	29 ± 3	0.91 ± 0.01
cassava	C_0_	4.7 ± 0.4	1.05 ± 0.01				
	C_10_	5.3 ± 0.5	1.10 ± 0.01				

a The errors represent the standard deviation of the average of at least two independent replicates.

TPS/PVA samples slightly increased their OTR at C_10_. This evolution may be due to the light degradation in terms of molecular weight reduction and caramelization along the reprocessing cycles, which lead to the formation of voids and channels, increasing the oxygen transmission, similarly as reported by Mellinas et al.^62^

Different effects were observed for commercial bioplastics. PLA slightly decreases the OTR at C_4_, very probably due to hydrolytic degradation, which may promote its hydrophilic character, contributing to the oxygen pathway blockage.^61–64^ By contrast, the oxygen permeability of PHB slightly increases, due to polymer chain scission, resulting in an increase in molecular mobility, as seen in the MFI results (Section 3.3, Figure 4), favoring the oxygen transmission.^62^

## 4 Conclusions

The present work is the first-ever dealing with the mechanical reprocessing of TPS/PVA blends, one of the compounds that is fully friendly in marine environments. The main conclusion of the present work is as follows.

- TPS/PVA blends retain integrity and mechanical performance without a significant decrease up to 10 cycles, indicative of the potential of promoting circular economy applications (such as packaging) with this ocean-friendly polymer. Contrarily, commercial PLA suffers a complete loss of toughness in cycle 4, and PHB a severe loss of properties after cycle 1 due to the intense degradation.
- The botanical origin of the starch produces slight differences in the evolution of the properties upon the reprocessing cycles. TPS/PVA based on potato starch (B-type crystallinity) progressively loses some plasticizer (glycerol) as well as partially degrades, which deals with a slight increase of the tensile modulus up to cycle 5, mainly due to the effect of losing plasticizer. Later, there is a slight decrease in stiffness when degradation becomes more apparent, reducing the influence of the retrograded amylose phase. On the contrary, wheat starch (A-type crystallinity) based TPS/PVA yields stable values along 10 cycles, forming a more plasticized and stable structure during more reprocessing cycles. This is because there is initially a low accessibility of the plasticizer to the grains, due to the low external diffusion to the internal pores, without reaching complete plasticization, which is advancing along the cycles, thanks to the more effective distribution of the plasticizer. Consequently, wheat-based ones can be reprocessed for more cycles. Cassava starch (C-type crystallinity) TPS/PVA presents an intermediate recycling potential compared to the other starches, but closer to wheat-based ones.
- Therefore, these TPS/PVA blends not only provide an ocean-friendly bioplastic but also can extend their useful life thanks to their higher potential recyclability and mechanical stability, remaining longer in a closed cycle of the circular economy and helping to achieve the objectives 12–15 of the Sustainable Development Agenda 2030.

## Supplementary Material



## Notes

Starch granule size and dispersion results by laser diffraction; and starch granule differential porosimetry by mercury porosimetry (PDF)
