# Supplementary material for: Mechanical Recyclability of TPS/PVA Blends and Their Comparison with Other Bioplastics
Source: ACS Omega. 2026 Mar 6;11(10):15765–76. doi: 10.1021/acsomega.5c06947 (PMC13000638; doi:10.1021/acsomega.5c06947)
Supplement: Supplementary file 1 [file ao5c06947_si_001.pdf]

MECHANICAL RECYCLABILITY OF TPS/PVA BLENDS AND ITS COMPARISON WITH OTHER BIOPLASTICS.

Noelia Martínez-Pérez, Juan C. García Quesada, Ignacio Martín-Gullón and Iluminada Rodríguez-Pastor.*

Institute of Chemical Process Engineering, University of Alicante

P.O. Box 99, 03080 Alicante, Spain.

Supporting Information

1. Potato, wheat and cassava starch granule size and dispersion results by Laser Diffraction.

Table S.1 shows the starch granule size determined by laser diffraction (LD). According to the average diameter, potato starch has the largest granule size by a considerable margin, followed by wheat starch and cassava starch. Based on the SPAN value, potato starch has the highest size dispersion, followed by wheat and cassava starch, which have very close dispersion values. These results are consistent with those of literature [1–3]. However, in some of these articles, cassava and wheat have higher size dispersion [2,3].

Table S.1. Particle size analysis of potato, wheat and cassava starches, by LD. The errors represent the standard deviation of the average of two independent replicates.

Botanical Origin	D (0.1) (µm)	D (0.5) (µm)	D (0.9) (µm)	Average Diameter (µm)	SPAN
Potato	24.7 ± 0.1	44.79 ± 0.01	77 ± 1	44.79 ± 0.01	1.17 ± 0.03
Wheat	12.3 ± 0.1	20.04 ± 0.07	32 ± 1	19.98 ± 0.01	0.96 ± 0.05
Cassava	8.6 ± 0.3	13.65 ± 0.06	21.02 ± 0.01	13.75 ± 0.08	0.91 ± 0.02

2. Potato, wheat and cassava starch granule differential porosimetry by mercury porosimetry.

Figure S.1 it can be noticed the distribution curves of mean pore diameters for the three starches studied for both interparticle (Figure S.1.A) and intraparticle pores (Figure S.1.B).

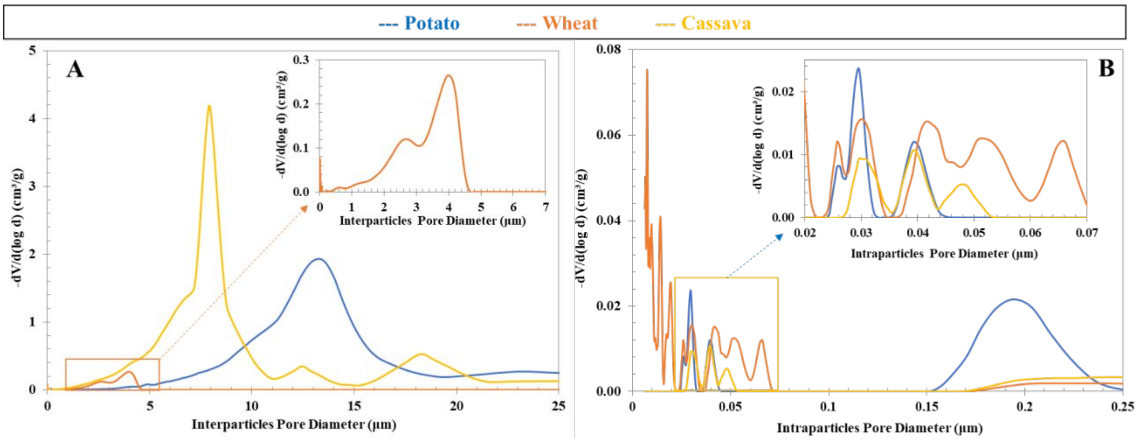

Figure S.1. Differential porosimetry curves for potato, wheat and cassava starches for interparticles (A) and intraparticles (B) pores.

REFERENCES

(1) Domene-López, D.; García-Quesada, J. C.; Martin-Gullon, I.; Montalbán, M. G. Influence of Starch Composition and Molecular Weight on Physicochemical Properties of

Biodegradable Films. *Polymers (Basel)* **2019**, *11* (7). https://doi.org/10.3390/polym11071084.

(2) Luchese, C. L.; Spada, J. C.; Tessaro, I. C. Starch Content Affects Physicochemical Properties of Corn and Cassava Starch-Based Films. *Ind Crops Prod* **2017**, *109*, 619–626. https://doi.org/10.1016/j.indcrop.2017.09.020.

(3) Luchese, C. L.; Benelli, P.; Spada, J. C.; Tessaro, I. C. Impact of the Starch Source on the Physicochemical Properties and Biodegradability of Different Starch-Based Films. *J Appl Polym Sci* **2018**, *135* (33). https://doi.org/10.1002/app.46564.
